# Does Ramadan fasting has any effects on menstrual cycles?

**Published:** 2013-02

**Authors:** Mahnaz Yavangi, Mohammad Ali Amirzargar, Nasibeh Amirzargar, Maryam Dadashpour

**Affiliations:** 1 *Department of Obstetrics and Gynecology, Fatemieh Infertility Research Center, Hamadan University of Medical Sciences, Hamadan, Iran.*; 2 *Department of Urology, Hamadan University of Medical Sciences, Hamadan, Iran.*; 3 *Tehran University of Medical Sciences, Tehran, Iran.*; 4 *Hamedan University of Medical Sciences, Hamedan, Iran.*

**Keywords:** *Fasting*, *Menstruation*, *Menstrual cycle*, *Binge eating disorder*, *Leptin*

## Abstract

**Background:** During the month of Ramadan, millions of Muslims abstain from food and drink daily from dawn to sunset and people actually experience repeated cycles of fasting and refeeding. Menstruation is a normal physiological process that its regularity is controlled by hypothalamic-pituitary-ovarian axis. Etiology of menstrual dysfunction includes weight loss, hypoleptinemia, abnormal eating behaviors, exercise, and psychological stressors.

**Objective:** To investigate the effects of Ramadan fasting on menstrual cycles.

**Materials and Methods:** This analytic cross-sectional study was performed on 80 female college students resident in a dormitory of Hamedan University of Medical Sciences. A questionnaire including demographic characteristics and menstrual calendar was filled by all participants. All analyses were performed using the statistical software SPSS for Windows version 11.5.

**Results:** We found 11.3%, 30%, and 16.3% of participates had abnormal menstrual pattern three months before, during and three months after Ramadan, respectively. In participates who fast more than 15 days, menstrual period had significantly more abnormality than participants who fast less than 15 days. Considering our results we demonstrated that menstrual abnormalities during Ramadan month reach to their peak and three months after Ramadan reduce but do not return to previous condition.

**Conclusion:** This study confirms that menstrual abnormalities including oligomenorrhea, polymenorrhea and hypermenorrhea increased during Ramadan especially in participates with more than 15 days of fasting.

## Introduction

During the month of Ramadan it is estimated that, worldwide, millions of healthy adult Muslims are required to abstain from food and drink from dawn to sunset daily which in the year 2009 runs from 22 August to 19 September. This is an experience of repeated cycles of fasting-refeeding. Naturally, fasting causes significant changes in the normal feeding, sleeping and behavioral patterns of the people. 

However, there have been only a few studies that examined the effect of the fast on basic homeostatic functions. Recently, medical studies have focused on the clinical effect of fasting on healthy people and its risks to patients with systemic disease. Considering the menstrual cycle, plays a significant role in women's health and disease, evaluation the effect of Ramadan fast on it seems necessary. 

Menstrual cycle is generally defined in the context of four dimensions of cycle regularity, menstrual period frequency, and duration of flow and volume of blood loss (1). The mean menstrual cycle length in the mid-reproductive years is between 28 and 30 days. The duration of the period of menstruation is commonly 5 days, whereas the volume of blood loss varies between 25 and 35 ml (2). An endocrine axis functions via hormonal regulation and feedback loops govern the regulation of menstruation known as the hypothalamic-pituitary-ovarian (HPO) axis. A variety of conditions affect this axis manifests as a final common symptom; irregular menstrual cycles (3). 

The majority of women reported at least one menstrual cycle classified as irregular, and about 37% reported both short and long cycles (4, 5). College-age young women frequently experience a variety of menstrual-related complaints, including dysmenorrhea, menorrhagia, irregular menses, and menstrual-related mood changes. The prevalence of menstrual dysfunction among college students has been reported to be from 13.9-21%. (6).

Irregular menstrual cycles may cause diabetes type 2, cardiovascular disease, osteoporosis and infertility and also some factors such as smoking, exercise, diets, body mass index (BMI), menarche`s age and the duration of menstruation and psychosocial stress can be associated with irregular menstrual cycles (2, 7-13). 

To our knowledge, this is the first study carried out to explore the effect of Ramadan fast on menstrual cycles in female college students. We aimed to compare the menstrual period pattern before, during and after Ramadan and investigate confounding factors affect cycle regularity.

## Materials and methods

This analytic cross-sectional study was designed according to a protocol approved by the ethical committee of Hamedan University of Medical Sciences. The female college students who lived in the dormitory of Hamedan University of Medical Sciences were asked to volunteer to take part in our study. The study was conducted from 22 June to 19 December of 2009, 3 months precedent to 3 months following Ramadan. 

Convenience sampling was performed. Dispensation from fasting is allowed during sickness, menstruation, pregnancy, breast feeding, and travel. Subjects who were unable to or not recommended to fast were excluded. The students who wanted to fast during 3 months before Ramadan were not included. Consumption of medications affecting sexual hormones was considered as exclusion criteria. Sampling was based on the number of all available cases in our target center. Eighty students of the dormitory based on the inclusion and exclusion criteria wanted to enroll in the study. After explaining the purpose and methods of our study, written informed consent was obtained from each participate.

At the beginning of investigation, detailed medical histories of the participants were taken via a questionnaire including demographic characteristics and menstrual calendar. All subjects underwent physical examination 3 months prior to fasting. Information on body mass (kg) and stature (m) were obtained from all participants and BMI was calculated. Patients with BMI exceeding 30 were considered obese. BMI levels were based on the WHO classification of obesity (14). Participates encourage to record their first date of each menstrual period during the 7 months of study. We attempt to measure menstrual blood loss on the basis of number of pads or tampons used per day or frequency of pad changes. At the end of the study questionnaires were collected and BMI was calculated again.

**Statistical analysis**

All analysis were performed using the SPSS software for Windows version 11.5. Analysis were done by nonparametric Chi-square test to evaluate the distributions. 

## Results

After collecting the questionnaire from 80 college students resident in the dormitory of University of Medical Sciences, we found that 11.3%, 30%, and 16.3% of participates had irregular menstrual pattern before, during and after Ramadan, respectively (Table I). P-value for menstrual pattern between these times were 0.0001 that indicate significant probability distribution of individuals. Mean age of participates was 24.7±3.1 years (range: 18-30 yrs).

To evaluate the effect of the duration of the fast on the menstrual pattern, it was seen that during 13 participates who fast less than 15 days, abnormal menstruation was 0%, 22.1% and 7.7% before, during and after Ramadan, respectively. These data among 67 participants who fast more than 15 days were 13.5%, 31.3% and 17.9%, respectively (Table II). With regard to lower number of whom fast less than 15 days in contrast with whom fast more than 15 days, the p-value of 0.0001 between groups did not have enough accuracy. In the group who fast less than 15 days the only abnormality which remains after Ramadan was oligomenorrhea. Among those who fast more than 15 days, the abnormality which remains more after Ramadan was polymenorrhea. Considering BMI characteristic, 80% of participates had normal weight, 17.5% were lean, and 2.5% were fat. In lean group normal menses decreased to 78.6% during Ramadan. More frequent abnormality was oligomenorrhea (Table III). In this group p-value for comparison pre Ramadan with Ramadan was 0.082 and for comparison during Ramadan and post Ramadan was 0.999 which means distributions were not significant. Among normal weight group menstrual abnormality were 12.5%, 31.2% and 18.7% before, during and after Ramadan, respectively. The p-value of 0.0001 in this group means significant differences between these periods. Hypermenorrhea was the most common abnormalities during Ramadan in this group (Table III). From 2 fat participates, one had hypermenorrhea in 7 months duration of the study (Table III). Considering that only 3 cases (3.8%) of participates had professional exercise activity and no one smoked, we did not statistically analyzed these two factors. 

**Table I:** Menstrual pattern in students before, during, and after Ramadan

**Menstrual pattern **	**3 months before Ramadan** **n(%)**	**During Ramadan** **n(%)**	**3 months after Ramadan** **n(%)**
Normal menstruation	71 (88.7)	56 (70)	67 (83.8)
Oligomenorrhea	1 (1.3)	11 (13.8)	2 (2.5)
Polymenorrhea	5 (6.3)	7 (8.8)	8 (10)
Hypermenorrhea	3 (3.8)	6 (7.5)	3 (3.8)
Summation	80 (100)	80 (100)	80 (100)

**Table II:** Menstrual pattern in students before, during, and after Ramadan considering the duration of fast

** Duration of fast**	**Less than 15 days ** **n(%)**				**More than 15 days** **n(%)**		
**Menstrual pattern**	**3 months before Ramadan**	**During Ramadan**	**3 months after Ramadan **		**3 months before ** **Ramadan**	**During Ramadan**	**3 months after Ramadan**
Normal menstruation	13 (100)	10 (76.9)	12 (92.3)		58 (86.5)	46 (68.7)	55 (82.1)
Oligomenorrhea	0 (0)	1 (7.7)	1 (7.7)		1 (1.5)	10 (14.8)	1 (1.5)
Polymenorrhea	0 (0)	1 (7.7)	0 (0)		5 (7.5)	6 (9)	8 (11.9)
Hypermenorrhea	0 (0)	1 (7.7)	0 (0)		3 (4.5)	5 (7.5)	3 (4.5)
Summation	13 (100)	13 (100)	13 (100)		67 (100)	67 (100)	67 (100)

**Table III:** Menstrual pattern in students before, during, and after Ramadan considering their BMI

**Menstrual pattern**	**3 months before Ramadan** **n(%)**				**During Ramadan** **n(%)**				**3 months after Ramadan** **n(%)**		
	**Lean**	**Normal**	**Fat**		**Lean**	**Normal**	**Fat**		**Lean**	**Normal**	**Fat**
Normal menstruation	14 (100)	56 (87.5)	1 (50)		11 (78.6)	44 (68.8)	1 (50)		14 (100)	52 (81.3)	1 (50)
Oligomenorrhea	0 (0)	1 (1.6)	0 (0)		2 (14.3)	5 (7.8)	0 (0)		0 (0)	2 (3.1)	0 (0)
Polymenorrhea	0 (0)	5 (7.8)	0 (0)		1 (7.1)	6 (9.4)	0 (0)		0 (0)	8 (12.5)	0 (0)
Hypermenorrhea	0 (0)	2 (3.1)	1 (50)		0	9 (14)	1 (50)		0 (0)	2 (3.1)	1 (50)
Summation	14 (100)	64 (100)	2 (100)		14 (100)	64 (100)	2 (100)		14 (100)	64 (100)	2 (100)

## Discussion

In this study for the first time we assessed the effect of Ramadan fast on menstrual cycles in female college students. The volunteers who take part in our trial were chosen from students who lived in the dormitory of University of Medical Sciences, which causes the similarity of confounding factors affecting menstrual pattern especially diet, stress and environmental factors such as light. Considering our results we demonstrated that menstrual abnormalities during Ramadan month reach to their peak and three months after Ramadan reduce but do not return to previous conditions. Besides, among participates who fast more than 15 days menstrual period had more abnormalities than volunteers who fast less than 15 days.

Menstruation is a normal physiological process that its regularity is controlled by action and interaction of hormones released from hypothalamus, pituitary and ovaries and their effect on the endometrium. In normal menstrual pattern length of menstrual cycle has an average of 28.1 days, length of flow is 3-7 days and amount of flow is <80 mL. Common menstrual disorders include heavy flow (hypermenorrhea), unusually light (hypomenorrhea), unusually frequent (polymenorrhea), unusually infrequent (oligomenorrhoea) and unusually painful (dysmenorrhea) (13). 

The frequency of irregular cycles ranged from 5-16% (14). The etiology of menstrual dysfunction includes weight loss, hypoleptinemia, abnormal eating behaviors, exercise, and psychological stressors (15). Oligomenorrhea was reported greater with binge eating, vomiting, and appetite suppressant use (16). Fasting or excessive weight loss can inhibit cycling and ovulation (17). Diet through modification of electroencephalographic activity and sleep patterns or by direct action alters hormone metabolism (18, 19). 

The fast entails absolute abstention from allowing anything to enter the body during the hours from dawn to sunset. Aside from being used as medical treatment for several conditions, fasting is also a spiritual activity for several religions, such as Islam, Christianity, Buddhaism, and Judaism. The Ramadan fast is required of every healthy adult Muslim for a complete lunar month (approximately 15 hours/day in Iran on 2009). The Ramadan may have several effects on basic homeostatic functions. 

During the month of Ramadan, people actually experience repeated cycles of fasting and refeeding. In addition there is a significant change in the daily patterns of behavior such as sleeping pattern (20). On average, people wake up earlier in the morning before sunrise for the first meal during the month of Ramadan. Many (65%) return to sleep later, fast for the rest of the day and some add an afternoon nap. These behavioral changes might cause alterations in hormonal secretion. During the fasting days of Ramadan glucose homeostasis is maintained by meals taken before dawn and by liver glycogen stores. 

Fasting in Ramadan is safe for the majority of diabetic patients with proper education and management (21, 22). Blood pressure does not change significantly during the Ramadan fast in patients who continue their daily medications (20). There are no adverse effects of Ramadan fasting on the heart, lung, liver, kidney, eyes, hematologic profile, endocrine and neuropsychiatric functions (23). Changes in serum lipids depend on weight and the quality and quantity of food consumption. Average body weight changes during the Ramadan month. Some people actually gained weight while others lost weight. Changes in BMI prior to, at the end, and after Ramadan influence by calorie, protein, and fat intake (24). 

In our study, participates were similar in considering confounding factors such as BMI and physical activity. In a cross-sectional study, analysis of 265 university students showed that more people got involved in stress reducing (watching TV, listening to the radio and visiting) and spiritual activities (prayers and reading Quran) during Ramadan. Food intake appeared to improve during this month with higher proportions eating foods from all food groups. The amount of foods did not differ significantly except in the case of foods from the cereal, meat and vegetable groups. They drank less caffeine-containing beverages and smoked less (25). In our study no cases smoke cigarette. But considering that smoking cessation might be useful in reducing the prevalence of menstrual symptoms and cycle disorders, we predict that fasting during Ramadan which causes reducing the amount of smoking can have important effect on menstrual cycles (26).

Reduction of food consumption may cause activations of multiple metabolic and neuroendocrine changes resulting in amenorrhea (27, 28). Considering our study was the first investigation about the effect of fast on menstrual cycles there was no similar study to compare our results and discuss on conclusions. But a similar condition with fasting is seen in patient with binge eating behavior which is defined as eating an unusually large amount of food (29).

Studies demonstrate that the ingestion of a single large daily meal results in metabolic consequences, including increased fasting glucose levels, increased insulin responses and modulation of the leptin diurnal rhythm (30). Binge eating is hypothesized to have effects on the HPO axis (31, 32). Serum leptin levels demonstrate a diurnal rhythm with a peak in the middle of the night (33). Fasting results in a gradual decline in leptin levels, first observed after 6-8 h (34). Leptin can affect the HPO axis by acting directly on the hypothalamus and increase luteinizing hormone releasing hormone release, on the pituitary gland and stimulates LH and follicle-stimulating hormone (FSH) release, and on the gonads and stimulates steroidogenesis (35, 36). 

Short term fasting for 3 days in the midfollicular phase of the menstrual cycle decreased the number of LH pulses and during midluteal phase augmented 24-h pulsatile growth hormone (GH) secretion, increased mean serum cortisol, and decreased 24-h mean serum leptin concentrations, insulin, and insulin-like growth factor-I (IGF-I) concentrations (37). Prolonged restriction of food consumption can inhibit reproduction in humans (38). Chronic illness and anorexia nervosa with inducing hypothalamic amenorrhea can decrease serum leptin concentrations (39). Regarding our results, we suggest more evaluations on hormonal and endocrinal changes during Ramadan fast in more populated samples.

Considering the less amount of participates, comparing the effect of fast on different pattern of body weight was impossible because 80% of volunteers had normal weight. This can be evaluated in future investigations. It seems that the effects of fast on menstrual cycles are manifest in some special groups. Assessment of such an issue by dividing patients into sub-groups should be considered in future studies.

## Conclusion

In conclusion, this study confirmed that menstrual abnormalities including oligomenorrhea, polymenorrhea and hypermenorrhea increase during Ramadan especially in participates with more than 15 days of fasting.
